# Ac/Ds transposition for CRISPR/dCas9-SID4x epigenome modulation in zebrafish

**DOI:** 10.1242/bio.059995

**Published:** 2023-06-27

**Authors:** Vanessa Chong-Morrison, Sarah Mayes, Filipa C. Simões, Upeka Senanayake, Dervla S. Carroll, Paul R. Riley, Stephen W. Wilson, Tatjana Sauka-Spengler

**Affiliations:** ^1^ University of Oxford, Weatherall Institute of Molecular Medicine, Radcliffe Department of Medicine, Oxford OX3 9DS, UK; ^2^ University of Oxford, Institute of Developmental and Regenerative Medicine, Department of Physiology, Anatomy and Genetics, Oxford OX3 7DQ, UK; ^3^ University College London, Department of Cell & Developmental Biology, London WC1E 6BT, UK; ^4^ Stowers Institute for Medical Research, Kansas City, MO 64110, USA

**Keywords:** Zebrafish, Ac/Ds, CRISPRi, Enhancer, Antisense

## Abstract

Due to its genetic amenability coupled with advances in genome editing, zebrafish is an excellent model to examine the function of (epi)genomic elements. Here, we repurposed the Ac/Ds maize transposition system to efficiently characterise zebrafish *cis*-regulated elements, also known as enhancers, in F0-microinjected embryos. We further used the system to stably express guide RNAs enabling CRISPR/dCas9-interference (CRISPRi) perturbation of enhancers without disrupting the underlying genetic sequence. In addition, we probed the phenomenon of antisense transcription at two neural crest gene loci. Our study highlights the utility of Ac/Ds transposition as a new tool for transient epigenome modulation in zebrafish.

## Teaser

**Summary:** The Ac/Ds transposition system, which enables the continuous expression of guide RNAs for CRISPR/Cas perturbation, is adapted to examine the epigenome in zebrafish.

## INTRODUCTION

Genomic transposition is an established approach for somatic and germline integration of DNA constructs in the zebrafish model. Tol2-mediated transposition is a reliable method for generating transgenic reporter lines in zebrafish (Kawakami, 2004), while Ac/Ds and MMLV (Vrljicak et al., 2016) are used less. The maize Ac/Ds system (McClintock, 1950) consists of two Ds (Dissociation) genetic elements and an Ac (Activator) transposase (Fedoroff et al., 1983). Ac/Ds transposition led to highly efficient integration of reporter constructs with a remarkable germline transmission rate in the zebrafish (Emelyanov et al., 2006). It was subsequently used to generate the chemically inducible LexPR transactivation system (Emelyanov and Parinov, 2008; Kenyon et al., 2018), and perform systematic mutagenesis gene-trapping screens (Quach et al., 2015). While these studies demonstrated the utility of Ac/Ds as an efficient method for the propagation of transgenes through the germline, they overlooked its strong potential for transient expression of DNA elements in F0 embryos. Regardless of the integration method, transposition in F0 zebrafish embryos produces variable somatic results with high rates of non-specific background and mosaic expression, hence limiting the potential of this model for transient studies. Therefore, the analysis of exogenous features often relies on generation of transgenic F1 offspring, which is time- and resource-consuming for medium to high throughput screening of transgenes. We sought to develop a flexible molecular toolkit by repurposing the Ac/Ds system to transiently screen reporter constructs and target gene expression in the zebrafish with functional and quantifiable output.

Perturbation approaches in F0 zebrafish embryos currently exist, such as morpholino-mediated obstruction of protein synthesis or RNA-splicing or gene editing using TALENs or CRISPR/Cas tools. CRISPR/dCas9-based interference (CRISPRi) uses nuclease-deficient Cas9 (dCas9) targeted to specific genomic regions using guide RNAs (sgRNAs). dCas9 is targeted to transcription start sites (TSS) of genes to inhibit RNA Polymerase II by steric hindrance (Qi et al., 2013), or fused to effector domains such as Kruppel-associated box (KRAB) (Gilbert et al., 2013) or four concatenated mSin3 repressive domains (SID4x) (Konermann et al., 2013) to induce chromatin changes inhibitive of transcription. Crucially, this allows the tuning of gene expression without modifying the endogenous locus sequences (Liu et al., 2017; Thakore et al., 2015; Williams et al., 2018; Dong et al., 2017; Long et al., 2015). Unlike the creation of indels in Cas9 F0 mutants, where genome editing occurs early in development and is propagated through subsequent cell divisions, CRISPRi in F0 embryos requires extended expression of sgRNAs for the duration of an experiment. This is limited by the current gold standard approach of injecting *in vitro*-transcribed sgRNAs, which degrade quickly in the absence of Cas9 protein (Burger et al., 2016; Hendel et al., 2015). Reliable assessment of CRISPRi effects will therefore be limited to early development in F0 embryos and require germline propagation of sgRNA constructs for later-stage analyses.

In this study, we demonstrated that Ac/Ds transposition is an efficient method for the transient propagation of transgenes with sustained expression of sgRNAs to 5 dpf. As a result, Ac/Ds-integrated sgRNAs in microinjected transgenic embryos expressing *sox10*-specific dCas9-SID4x enabled tissue-specific perturbation of epigenomic features in the zebrafish with robust and detectable effects from 24 hpf. Our approach broadens the utility of the zebrafish embryo for rapid studies of non-coding genomic elements, including enhancers, and complements current methods for targeting protein-coding genes.

## RESULTS AND DISCUSSION

### Ac/Ds transposition enables efficient expression of transgenes in F0 embryos

**Fig. 1.**
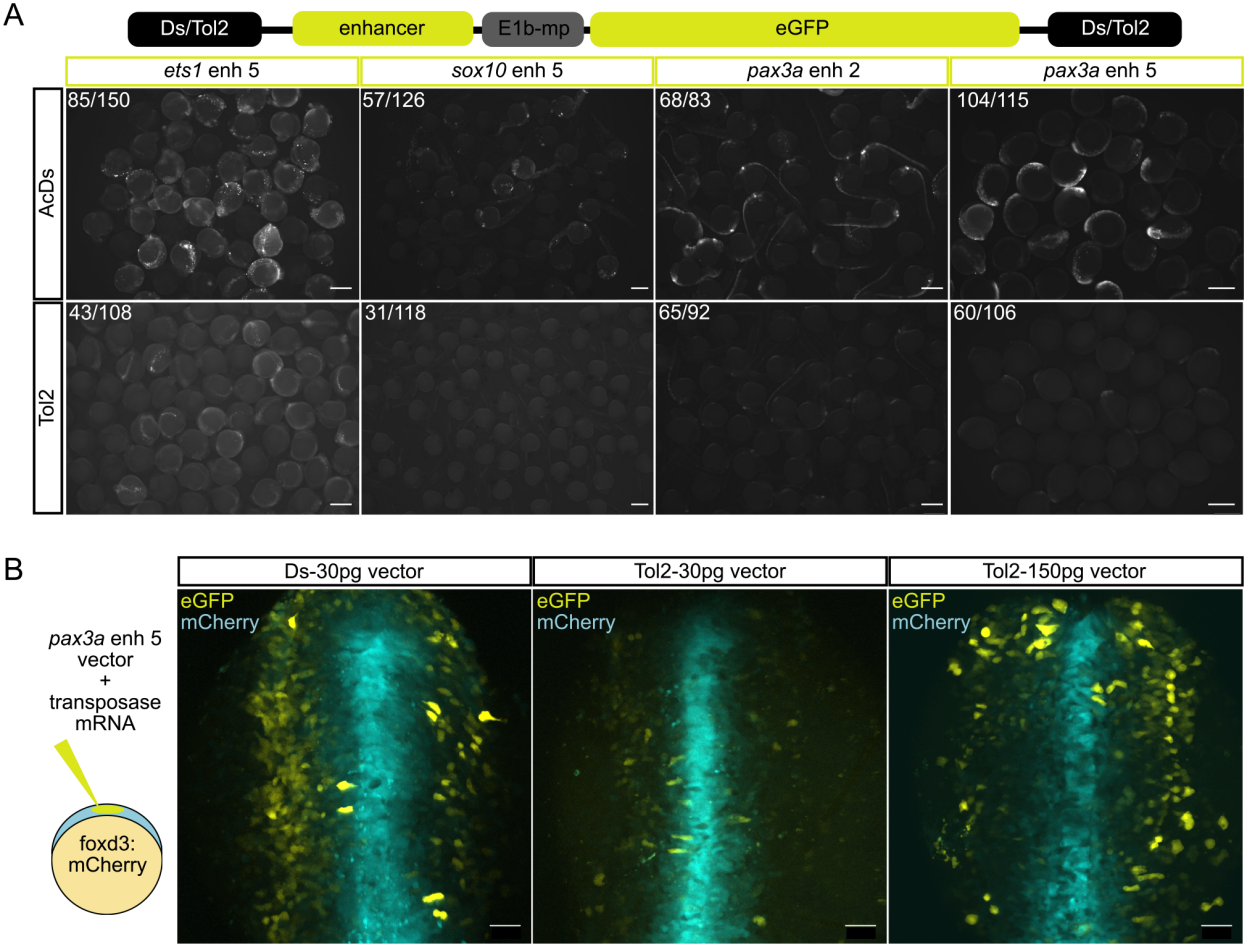
**Evaluation of Ac/Ds and Tol2 transposition in F0 embryos**. (A) Schematic of enhancer construct containing enhancers (‘enhancer’) upstream of E1b minimal promoter (‘E1b-mp’) driving eGFP expression. Two versions of each enhancer construct (with Ds- or Tol2-integration arms) were tested by microinjection into one-cell-stage embryos. Ac or Tol2 clutches of the same enhancer were imaged using the same settings on a fluorescent stereomicroscope. Embryos with the same expression pattern (Table S1) were counted. Scale bars: 500 µm. (B) Ds- or Tol2-armed *pax3a enh5* was microinjected with Ac or Tol2 mRNA, respectively, into Gt(FoxD3:mCherry)^ct110aR^ embryos to visualise the neural tube (mCherry, cyan). Live confocal imaging highlighted similar levels of neural crest cell labelling (eGFP, yellow) between 30 pg Ds and 150 pg Tol2 vector DNA, but 30 pg Tol2 vector DNA yielded a visually weaker signal. Scale bars: 50 µm.

To repurpose the maize Ac/Ds transposition system for zebrafish, we first generated a new enhancer–reporter construct *pVC-Ds-E1b:eGFP-Ds* (‘Ac/Ds-enh’). A multiple cloning site for testing enhancer sequences was placed upstream of an E1b minimal promoter (Becker et al., 2016), driving the expression of eGFP. The entire cassette was flanked by Ds elements for integration into the genome (Emelyanov and Parinov, 2008; Emelyanov et al., 2006) (Fig. 1A). We compared the activity of Ac/Ds- and Tol2-mediated transposition (Kawakami, 2004) by transiently expressing previously identified zebrafish enhancers for *pax3a*, *ets1* and *sox10* (Lukoseviciute et al., 2018). Embryos were microinjected with the same amounts of nucleic acid (30 pg Ac/Ds or Tol2 vector DNA, 24 pg Ac or Tol2 mRNA), and those with the same expression patterns in both conditions were counted. We found that Ac/Ds is similar or better than Tol2 in producing embryos with specific expression patterns (45.2 to 88.0% versus 26.3 to 75.0%) for the six different enhancers we have tested (Fig. 1A; Table S1). Tol2 integrations consistently exhibited a fainter eGFP signal which can be bypassed by injecting higher amounts of nucleic acid (150 pg vector DNA with 80 pg mRNA) (Fig. 1B).

The high efficiency of Ac/Ds integrations with lower amounts of DNA and mRNA renders it an excellent transient DNA expression system in zebrafish. Not only can the toxicity issues be avoided (those are observed when higher injected levels are required for somatic integration), but ectopic activity from the episomal expression of the non-integrated plasmid is also minimised. This places the zebrafish embryo on par with the chick embryo as an excellent model for testing enhancer activity transiently in F0 embryos (Streit et al., 2013; Weinberger et al., 2021 preprint). We conclude that our Ac/Ds transposition approach enables consistent tissue-specific expression patterns and is an excellent binary tool for transient screening of enhancer activity in zebrafish.

### Ac/Ds-sgRNA expression system for transient CRISPR/Cas

**Fig. 2.**
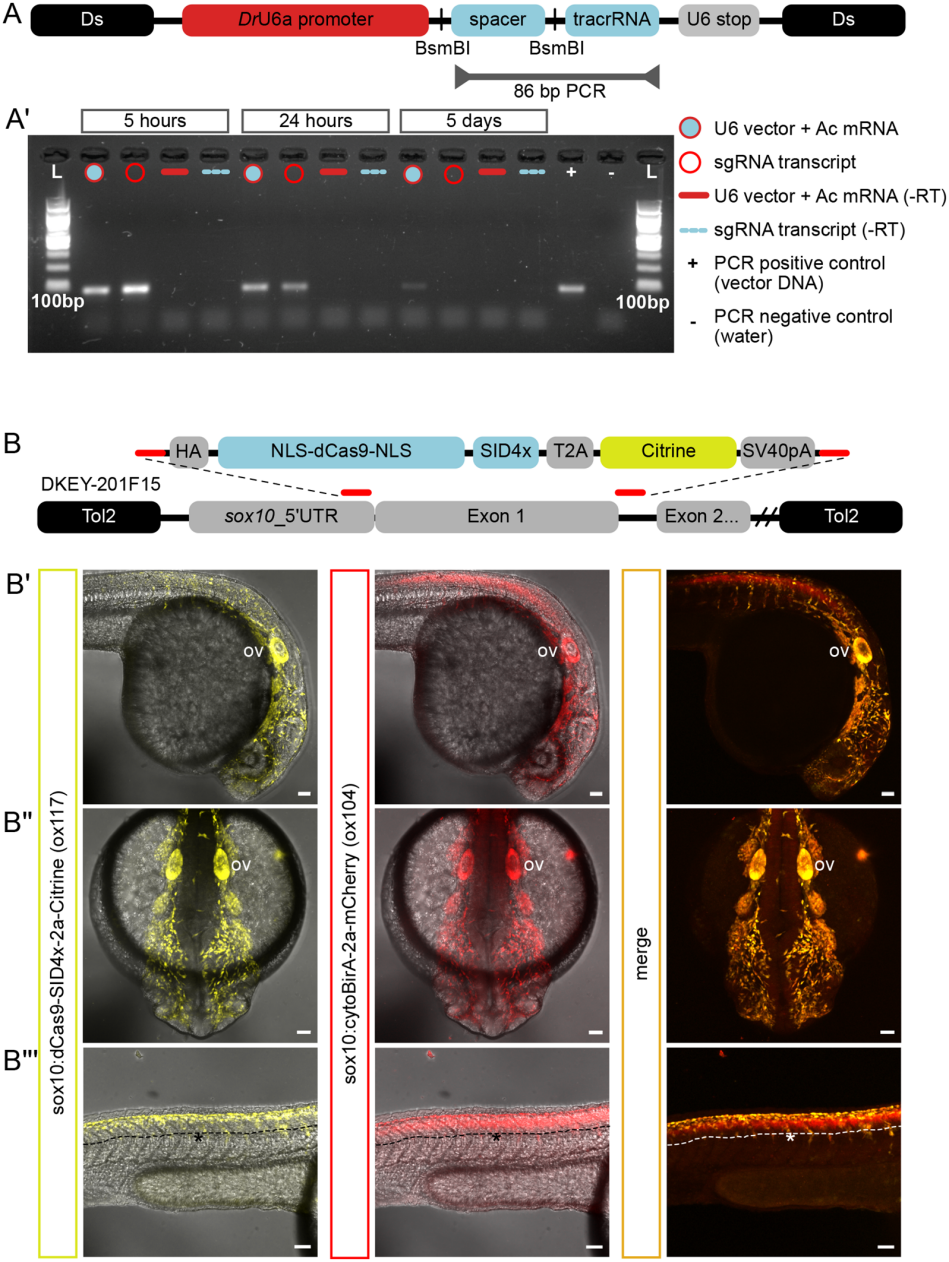
**Components of a CRISPRi genetic toolkit in zebrafish.** (A) Schematic of Ac/Ds construct containing a guide RNA region (‘spacer’ and ‘tracrRNA’) cloned downstream of the zebrafish U6a promoter (‘*Dr*U6a promoter’) followed by a U6 polymerase termination sequence (‘U6 stop’). The ‘spacer’ region is flanked with BsmBI restriction sites for Golden-Gate-like cloning of target spacers-of-choice. To evaluate expression of a scrambled guide RNA, RT-PCR primers and conditions were optimised to produce an 86 bp amplicon spanning the spacer and tracrRNA. (A′) RNA was collected from embryos injected with either the U6 vector scrambled sgRNA (filled circle) or *in vitro*-transcribed sgRNAs (empty circle) at 5 h, 24 h and 5 days post-injection. Only the RT-PCR product from U6-sgRNA injections could be detected at 5 days. (B) Schematic of BAC recombination to generate a Sox10-specific CRISPRi transgenic line, *TgBAC(sox10:dCas9-SID4x-2a-Citrine)^ox117^*. A recombination cassette contains nuclease-deficient Cas9 (‘NLS-dCas9-NLS’) fused to the SID4x repressor domain (‘SID4x’) followed by a ribosome-skipping Tav-2a peptide (‘T2A’) and Citrine fluorescent protein (‘Citrine’). Homology arms (red lines) enabled replacement of *sox10*’s first exon in BAC clone DKEY-201F15 with the dCas9-SID4x cassette. Transgenic offspring displayed largely overlapping expression with a different allele made from the same BAC (*TgBAC(sox10:cytoBirA-2a-mCherry)*, ‘*ox104*’) in cranial and trunk neural crest cells, as well as the otic vesicle (ov) (B′,B″). However, unlike the *ox104* line, Citrine is not expressed in *ox117*’s neural tube (B‴). Scale bars: 50 µm.

Next, we capitalised on robust Ac/Ds transposition in transient to generate a constitutive sgRNA expression system for CRISPR/Cas experiments in F0 embryos. We cloned a sgRNA cassette containing a zebrafish-specific U6a promoter (Yin et al., 2015) into a custom-made Ac/Ds mini-vector (‘Ac/Ds-sgRNA’) (Fig. 2A). The 20 bp spacer region within the cassette was flanked by BsmBI restriction sites to facilitate Golden Gate-like cloning (Clarke et al., 2012) of different sgRNAs. We compared the expression of a scrambled sgRNA sequence (AGCGCCTACGCGCATGGCCT) from integrated Ac/Ds-sgRNA versus *in vitro*-transcribed transcript(s). The vector (50 pg) with Ac mRNA (24 pg), were microinjected into wild-type embryos and sgRNA expression was assayed 5 and 24 h post injection (hpi) and 5 days post injection (dpi). We found that sgRNA expression at 5 dpi could only be detected using Ac/Ds (Fig. 2A′). Consequently, we demonstrated in a separate study that our approach enabled efficient F0 mutant studies up to 5 dpf via co-injection with Cas9 mRNA (Weinberger et al., 2020).

To achieve tissue-specific CRISPRi, we generated a BAC transgenic line *TgBAC(sox10:dCas9-SID4x-2a-Citrine)^ox117^* (‘*ox117*’) using a previously validated sox10 BAC allele *TgBAC(sox10:cytoBirA-2a-mCherry)^ox104^* (‘*ox104*’) (Trinh et al., 2017). *Ox117* resulted from the replacement of sox10's first exon with a cassette containing dCas9 fused to four tandem mSin3 repressive domains (SID4x) (Konermann et al., 2013), T2A ribosome-skipping element, and Citrine fluorescent protein (Fig. 2B). Live imaging in *ox104* background revealed largely overlapping expression of the allele in cranial and trunk neural crest cells. Unlike *ox104*, dorsal neural tube expression was absent in *ox117* (Fig. 2B′), likely due to positional effect during genomic integration.

The ability to knockdown non-coding elements *in vivo* is essential for dissecting their function. While cost-effective and straightforward, microinjection of *in vitro*-transcribed sgRNAs into single-cell embryos risks decreasing the efficiency of CRISPR-mediated events as uncapped and non-polyadenylated sgRNAs are sensitive to degradation *in vivo* (Hendel et al., 2015). This is particularly pertinent if the desired goal is to perform CRISPR experiments in a tissue-specific fashion, as unprotected sgRNAs injected in the absence of Cas9 protein or Cas9 mRNA are likely to be diminished by the time a tissue-specific Cas9 protein is expressed later in development. As our Ac/Ds-sgRNA system bypassed this limitation, we reasoned that, in combination with *ox117* embryos, it is an ideal sgRNA delivery method to achieve sox10-specific CRISPRi (‘sox10:CRISPRi’).

### CRISPRi of endogenous enhancers induces detectable changes in gene expression

**Fig. 3.**
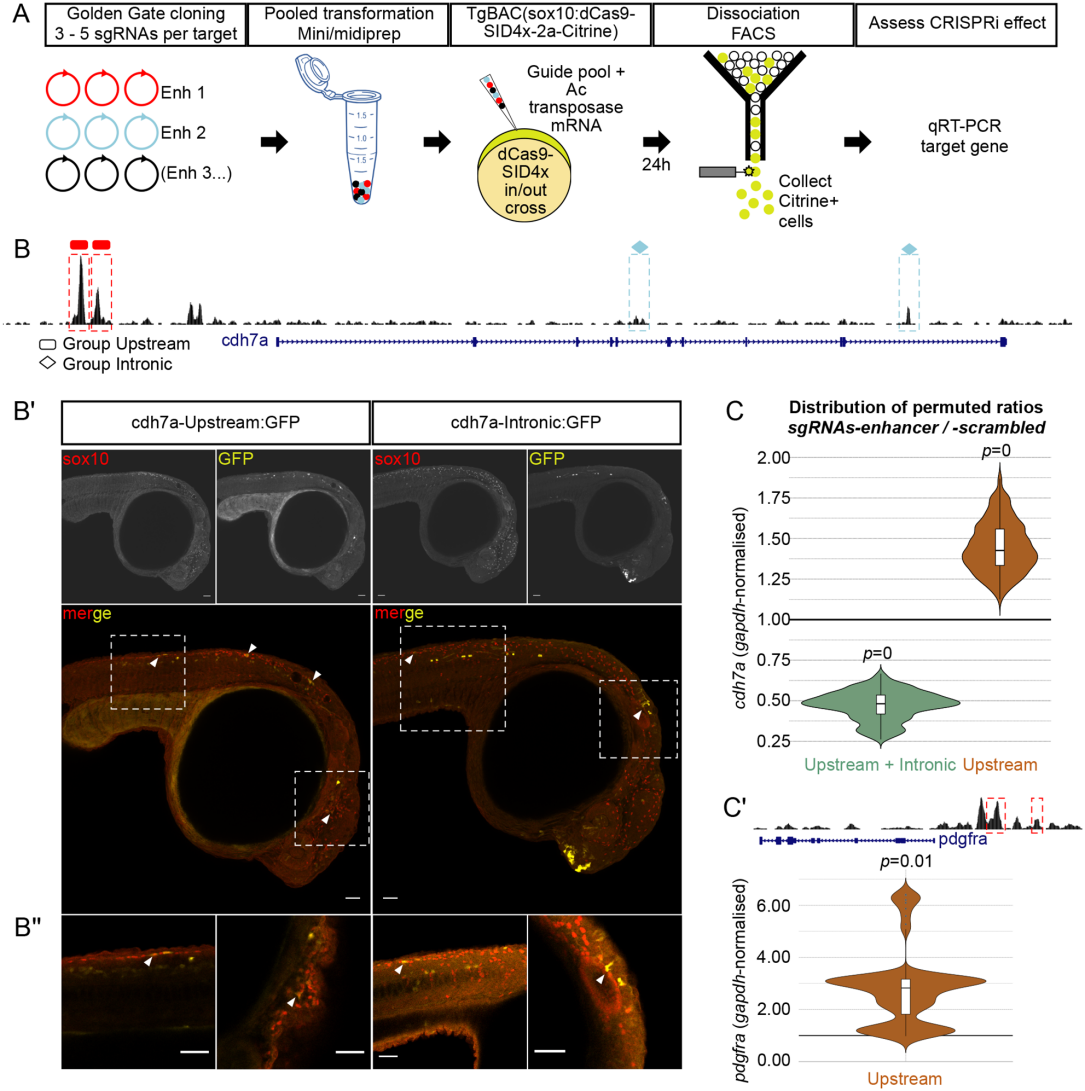
**CRISPRi of neural crest enhancers.** (A) Sox10:CRISPRi workflow to investigate function of enhancers in microinjected zebrafish embryos. 3-5 spacer sequences per enhancer for 2-4 enhancers per target gene were selected and individually cloned into Ac/Ds U6 vector. Cloned guides were combined according to experimental design and transformed to obtain a single prep per pool with sufficient quality and concentration. Scrambled guide pools were prepared in parallel as control. Guide pools (enhancer or scrambled) were microinjected into one-cell-stage *ox117* embryos and allowed to develop for 24 h. Embryos were dissociated and FAC-sorted to collect *sox10*:Citrine+ cells expressing dCas9-SID4x. RNA was extracted and expression of endogenous target controlled by the enhancers tested was measured by quantitative PCR. (B) UCSC Genome Browser snapshot of the *cdh7a* locus showing ATAC-seq from sox10+ cells (black track). Four *cdh7a* putative enhancer regions were investigated: two upstream (rectangles) and two within introns (diamonds). (B′) Combined readout of ‘upstream’ or ‘intronic’ *cdh7a* enhancer activity in F0 embryos. Each enhancer was cloned into Ac/Ds enhancer:GFP construct (Fig. 1). ‘Upstream’ or ‘intronic’ enhancers were pooled and pools microinjected into one-cell-stage embryos. Enhancer(s) activity (‘GFP’) in relation to endogenous sox10 (‘sox10’) expression was detected by immunohistochemistry at 24 hpf. (B″) Sporadic overlap (white arrows) of enhancer(s) activity were detected in the trunk neural crest in both cases, in the cranial mesenchyme only for ‘upstream’, and in the otic vesicle for ‘intronic’ enhancers. Maximum intensity Z-stack projections are shown in (B′), single plane confocal images are shown in (B″). Scale bars: 50 µm. (C) Quantitative PCR of *cdh7a* following CRISPRi of its putative enhancers (*n*=4 per condition). Downregulation of *cdh7a* (median±s.d.=0.481±0.089; *P*=0) was observed when all four enhancers were targeted, compared to scrambled control. This effect was reversed (median±s.d.=1.426±0.155; *P*=0) when the upstream enhancers only were targeted. (C′) A similar upregulation effect (median±s.d.=2.822±1.419; *P*=0.01) when upstream enhancers were targeted was observed at the *pdgfra* locus.

Previous studies have demonstrated the requirement for multiple sgRNAs to elicit successful CRISPRi effects (Qi et al., 2013; Williams et al., 2018). We reasoned that the small size of the Ac/Ds-sgRNA vector (<4.5 kb), coupled with the small load required for activity, would permit using multiple sgRNAs for CRISPRi microinjections. High-quality pools of individually cloned sgRNAs were prepared using a simplified pooled transformation approach reminiscent of the generation of large scale sgRNA libraries. *Ox117* transgenic embryos were microinjected with experimental (targeting putative enhancers) or scrambled sgRNA pools. Microinjection of 200 pg of sgRNA pool (consisting of 4 to 15 sgRNAs) per embryo yielded survival rates of ∼50% or higher. 24 hpf embryos were dissociated and FAC-sorted to obtain Citrine-positive cells expressing sox10 BAC-driven dCas9-SID4x. Gene expression was measured by TaqMan qPCR chemistry and qpcR analysis in R (Ritz and Spiess, 2008) (Fig. 3A).

We evaluated putative enhancers for cadherin 7a (*cdh7a*) gene. In chicken, Cdh7 function was first described in delaminating neural crest forming the melanocyte precursor subpopulation (Nakagawa and Takeichi, 1995, 1998), and later demonstrated to be important for trigeminal ganglia assembly (Wu and Taneyhill, 2019). We assessed four candidate zebrafish *cdh7a* enhancers (Up-1 and Up-2 i.e. ‘Group Upstream’; Int-1 and Int-2 i.e. ‘Group Intronic’) identified using chromatin accessibility data from zebrafish neural crest (Lukoseviciute et al., 2018; Trinh et al., 2017) (Fig. 3B). Using our Ac/Ds-enh vector we validated their activity in F0 embryos, where sporadic co-labelling with endogenous sox10 expression could be detected in the trunk neural crest and cranial mesenchyme (Group Upstream only) or otic vesicle (Group Intronic only) (Fig. 3B′-B″).

As these enhancers displayed detectable differences in chromatin accessibility in *foxd3*-mutants compared to wild type (Lukoseviciute et al., 2018), we reasoned that they were ideal for demonstrating utility of sox10:CRISPRi in investigating the function of novel enhancers. Targeting all enhancers simultaneously with a pool of 15 sgRNAs led to ∼50% downregulation (median±s.d.=0.481±0.089; *P*=0; *n*=4) of *cdh7a* expression compared to scrambled control (Fig. 3C, green plot). This was consistent with the additive action of multiple enhancers in regulating gene expression of their target genes (Choi et al., 2021; Fulco et al., 2016). Furthermore, this effect was reversed, with ∼50% upregulation of *cdh7a*, when sgRNAs targeting intronic enhancers were excluded from the pool (Fig. 3C, orange plot). Given that both groups of enhancers functioned cooperatively to fine-tune and maintain appropriate levels of *cdh7a* expression in scrambled sgRNAs/unperturbed condition, two plausible scenarios could explain our observation: either intronic elements acted as activator enhancers for *cdh7a* while upstream elements were repressive, or targeting dCas9-SID4x to Group Intronic may have inadvertently blocked transcriptional elongation of *cdh7a* itself. However, a previous study assessing steric hindrance effect of dCas9/dCas9-KRAB highlighted that this occurred when sgRNAs targeted −50 and +300 bp from the TSS of endogenous genes (Gilbert et al., 2014). Given that our enhancers were ∼20-72 kbp up or downstream of *cdh7a*’s TSS, we reasoned that the latter scenario was unlikely.

We further assessed two upstream enhancers of *pdgfra*, a previously identified gene with differential chromatin accessibility in vagal neural crest (Ling and Sauka-Spengler, 2019). We observed a similar effect to the *cdh7a* Group Upstream-only CRISPRi, with an upregulation of *pdgfra* expression following CRISPRi using a pool of 7 sgRNAs (median±s.d.=2.822±1.419; *P*=0.01; *n*=4) (Fig. 3C′). As these enhancers were proximal (both within ∼6.5 kb of the TSS), we speculate the presence of yet unidentified distal enhancer(s) with strong activating capacity akin to *cdh7a* distal Group Intronic enhancers (41 and 72 kbp downstream from TSS). Taken together, these results demonstrated the utility of our sox10:CRISPRi/Ac/Ds-sgRNA approach as an exploratory tool to transiently probe enhancer function *in vivo* in a cell-specific manner.

### CRISPRi at 5′ site of antisense transcription affects protein and chromatin homeostasis

**Fig. 4.**
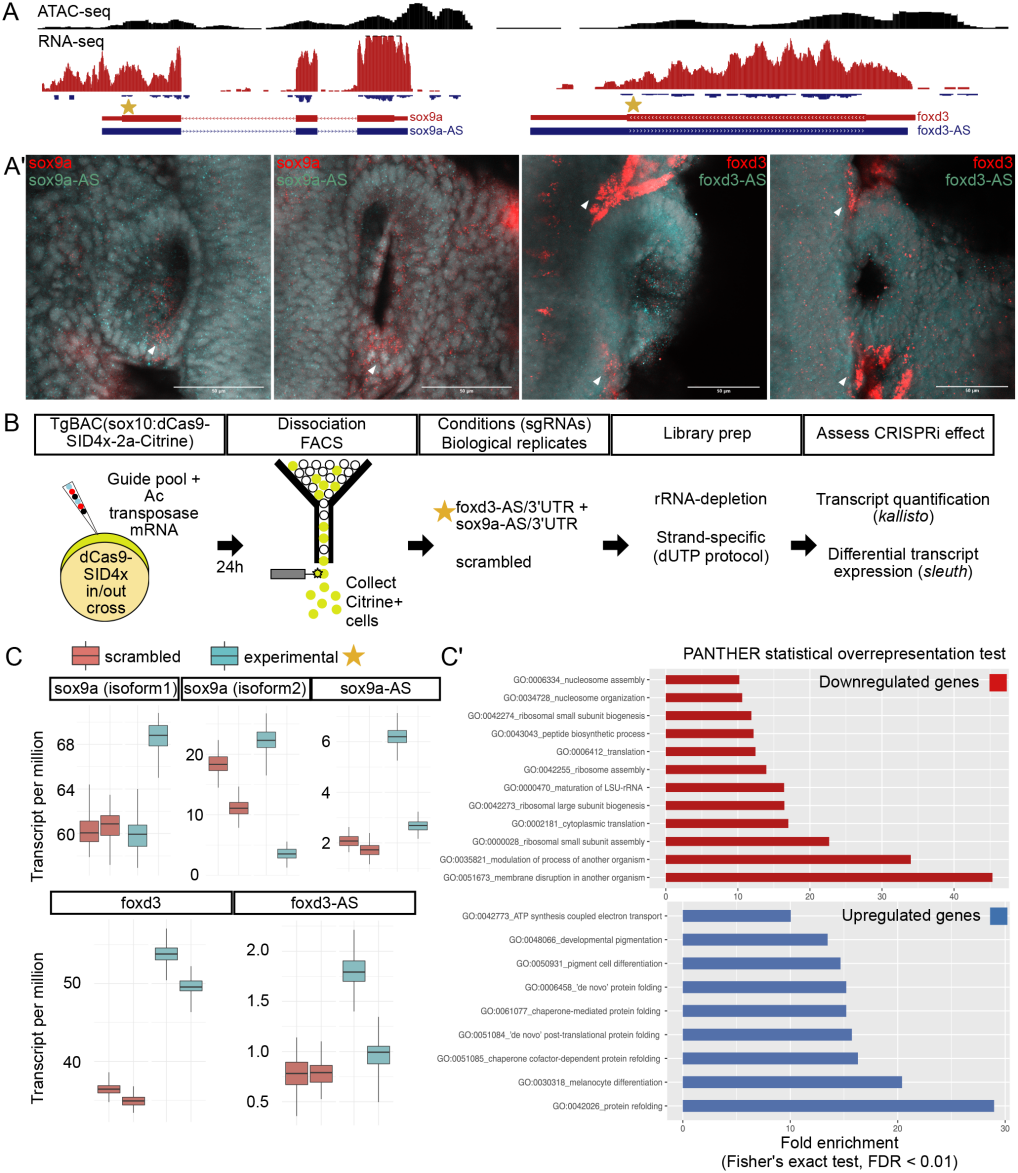
**CRISPRi of antisense transcription initiation at neural crest genes.** (A) UCSC Genome Browser snapshot of the *sox9a* and *foxd3* loci with ATAC-seq (black track) and strand-specific RNA-seq (red track, sense; blue track, antisense) from sox10+ cells (ATAC-seq) or nuclei (RNA-seq). Selected guide RNAs target the initiation of antisense transcription (star) of *sox9a-AS* or *foxd3-AS*. (A′) Hybridisation Chain Reaction detection of coding/sense (*sox9a*, *foxd3*) and antisense (*sox9a-AS*, *foxd3-AS*) transcripts in 24 hpf embryos. Antisense transcripts/puncta (cyan) were expressed in a ubiquitous, basal-like fashion. A similar basal-like expression of sense transcripts/puncta (red channel) was observed, along with spatially restricted regions of higher expression (white arrows). Single plane confocal images of the otic vesicle region are shown. Scale bars: 50 µm. (B) Sox10:CRISPRi workflow to investigate effect of sense/antisense transcription in microinjected zebrafish embryos. Ac/Ds U6 cloned guides were pooled according to experimental design (10 guides in total, five per locus) and transformed into bacteria to obtain a single prep per pool of high quality. Guide pools (foxd3+sox9a; or scrambled) were microinjected into one-cell-stage *ox117* embryos and allowed to develop for 24 h. Embryos were dissociated and FAC-sorted to collect *sox10*:Citrine+ cells expressing dCas9-SID4x. RNA was extracted and RNAseq libraries were prepared using rRNA-depletion followed by strand-specific dUTP method with two replicates per condition. Transcript quantification and differential expression were performed using the *kallisto*/*sleuth* statistical pipeline. (C) Quantification of transcripts (transcripts per million, TPM) following sox10:CRISPRi. Bootstrapped estimates of sense and antisense sox9a/foxd3 transcripts in each biological replicate. Down/upregulation could be detected for sense and antisense *foxd3*/*sox9a* transcripts in experimental versus scrambled condition although not statistically significant, with the exception of *foxd3* (FC=1.261, qval=0.007). (C′) PANTHER Gene Ontology (GO) overrepresentation statistical analysis of differentially expressed genes (qval <0.05). Top 10 GO terms (Fisher's exact test, with false discovery rate <0.01) shown.

CRISPRi with dCas9 alone was initially reported to have strand-specific activity when targeting transcriptional elongation, but not initiation, when using control sgRNAs that bind the non-template strand (Qi et al., 2013). However, this effect was less clear in later studies and was lost when dCas9 was fused to a repressor or activator domain (Gilbert et al., 2013, 2014; Howe et al., 2017). In light of these findings, we adapted sox10:CRISPRi/Ac/Ds-sgRNA to query antisense transcripts at two neural crest genes (*sox9a-AS* and *foxd3-AS*) (Fig. 4A). SgRNAs were selected irrespective of strand to target the initiation of antisense transcription (Fig. 4A, star). Wild-type staining and visualisation of *sox9a-AS* and *foxd3-AS* transcripts using hybridization chain reaction (Choi et al., 2018) indicated broad, low-level expression throughout the embryo, which partially overlapped their cognate genes, *sox9a* and *foxd3* (Fig. 4A′).

As before, *ox117* transgenic embryos were microinjected with a pool of 10 sgRNAs (five per locus) or four scrambled sgRNAs and FAC-sorted to obtain Citrine-positive cells expressing sox10 BAC-driven dCas9-SID4x. To assess local and global effects following CRISPRi, strand-specific RNAseq libraries were prepared, followed by unbiased transcript quantification and differential expression (Fig. 4B). Bootstrapped estimates of *sox9a-AS* and *foxd3-AS* indicated ∼2.3- and 1.7-fold upregulation (average, *n*=2), respectively, although neither was statistically significant (Fig. 4C). This lack of significance for the observed effect was unsurprising given their low levels of expression: *sox9a-AS* expression was detected ∼8- and 30-fold lower compared to its two cognate isoforms, while *foxd3-AS* expression was ∼50-fold lower. This result illustrated inherent challenges of capturing and studying lowly/dynamically expressed antisense transcripts in general. We also observed misregulation (up- or downregulation) of the sense transcripts, indicating a potential secondary effect on gene expression due to chromatin changes at their 3′UTR region resulting from dCas9-SID4x recruitment (Howe et al., 2017; Murray et al., 2015), which may have contributed to the net upregulation of *sox9a-* and *foxd3-AS* following CRISPRi.

Next, we asked if global cellular changes could be detected resulting from the observed misregulation in sense/antisense transcription of *sox9a* and *foxd3* (Fig. 4C′). Concomitant or concurrent with local destabilisation of *sox9a*/*foxd3* sense/antisense transcripts, we observed an overrepresentation of downregulated genes (qval<0.05) involved in nucleosome assembly and organisation, namely histone genes such as *h2ax, h2az2b, hist1h2a3, hist1h2a5, hist1h2a6, hist1h2a10, hist1h2a11, h3f3b.1, H3C15, h3f3d,* and *hist1h4 l*. This was consistent with reported dynamic histone turnover rates being correlated with the level of antisense, rather than sense, transcription (Murray et al., 2015). However, in our study it was not clear whether the effect was due to disruption of either or both. We also observed an overrepresentation of downregulated genes related to the translational machinery, including ribosomal genes such as *rpl15, rpl35a, rpl12, rps23, rps14, rpl39, rpl18, rpl8, rpl8, rpl21,* and *rps18*. On the other hand, protein-refolding genes, including chaperones, were upregulated (qval <0.05), as were genes involved in development of pigment cells, which are neural crest derivatives. Given the 3′UTR region's classical role in the translational regulation of mRNA transcripts, this was consistent with misregulation at the sense 3′ UTR regions of *sox9a* and *foxd3*. Taken together, we concluded that CRISPRi/Ac/Ds-sgRNA is a useful approach in zebrafish to study context-specific epigenome features and their impact on gene regulatory processes.

## MATERIALS AND METHODS

### Zebrafish husbandry

Zebrafish experiments were conducted according to regulated procedures authorised by the UK Home Office within the Animals (Scientific Procedures) Act 1986 framework. Wild-type and transgenic embryos were derived from AB or AB/TL mixed strains.

### Plasmids and oligo sequences

Plasmids (including Addgene IDs where applicable) and oligo sequences are available as Tables S2 and S3, respectively.

### Cloning of Ac/Ds-enh reporter vectors

Putative enhancer elements were amplified from genomic DNA by PCR and cloned into pVC-Ds-E1b:eGFP-Ds (Addgene ID 102417) linearised with NheI. Cloning was performed using In-Fusion HD Cloning Plus (Takara) or Gibson Assembly cloning (Gibson et al., 2009). 5′ overhangs were appended to enhancer-specific primers (Table S3) as follows: TCGAGTTTACGTACCGCTAG on forward and TATCGCCGCAAGCTTGCTAG on reverse.

Cloning reactions were transformed into chemo-competent cells and plasmids were prepared using QIAprep Spin Miniprep kit (27104, Qiagen) and stored in Elution Buffer. Preps were quantified using Nanodrop and inserts where verified by Sanger sequencing using T7 primer.

### Cloning of Ac/Ds-U6a:sgRNA vectors

We first generated a mini-vector with Ds arms flanking a multiple cloning site (pVC-Ds-MCS-Ds; Addgene ID 102416). An insert consisting of zebrafish U6a promoter, spacer region, tracrRNA, and U6 termination sequence (Yin et al., 2015) was amplified by PCR using a custom-ordered gBlock Gene Fragment (Integrated DNA Technologies) as template.

The PCR product was gel-purified and cloned into the mini-vector linearised with SnaBI and NheI, using In-Fusion^TM^ HD Cloning Plus (Takara). To clone desired sgRNAs, spacer sequences for targets-of-interest were selected using suitable algorithms e.g., CHOPCHOP (Labun et al., 2019), CRISPRscan (Moreno-Mateos et al., 2015), CRISPRz (Varshney et al., 2016), ensuring that they do not contain a U6 termination sequence (TTTT). Complementary oligo pairs were ordered as follows: (1) TTCG-5′[20 bp spacer without PAM]3′ and (2) AAAC-5′[20 bp spacer without PAM in reverse complement]3′ (Table S3). 50 µM of each oligo was combined in a 50 µl reaction and annealed in a thermocycler (94°C 5 mins, decrease to 22°C at 1°C/min, 4°C hold). Cloning reaction was prepared by combining 70 ng pVC-Ds-DrU6a:sgRNA-Ds vector, 5 ng annealed sgRNA, 10 U of BsmBI and 20 U of T4 DNA ligase (M0202, NEB) in 1X T4 DNA Ligase Buffer with final volume 20 µl. GoldenGate-like cycling conditions were used as follows: 10X(37°C 5 mins, 16°C 10 min), 50°C 5 mins, 80°C 5 mins. 2 µl of the reaction was transformed into chemo-competent cells and plated onto Ampicillin plates. One or two colonies per sgRNA were screened by Sanger sequencing using U6a promoter primer (TCACTCACCACCTCCCAAAA) and quantified using Nanodrop.

### Ac transposase mRNA synthesis

To prepare Ac transposase mRNA, pAC-SP6 (Addgene ID 102418) (Emelyanov et al., 2006) was linearised with BamHI and purified under RNase-free conditions. *In vitro* transcription was performed using mMESSAGE mMACHINE^TM^ SP6 Transcription Kit (AM1340, ThermoFisher). mRNA was purified under RNase-free conditions using phenol-chloroform followed by ethanol precipitation and the pellet resuspended in RNase-free water. mRNA quality was assessed by gel electrophoresis (sharp intact band without degradation) and quantified using Qubit^TM^ RNA HS Assay kit (Q32852, ThermoFisher). For long term storage in −80°C the purified mRNA was prepared as 1 µl aliquots and limited to one freeze-thaw cycle. Prior to use, an aliquot is freshly diluted with nuclease-free water.

### Ac/Ds microinjections

#### Preparation of Ac/Ds constructs/pools for microinjections

For long term storage, all stock preps were eluted in Elution Buffer (Qiagen). To minimise toxicity for microinjections, preps were diluted at least fivefold with nuclease-free water. Therefore, transformation efficiencies and elution volumes must be considered to maximise concentration of final preps.

#### Preparation of sgRNA-Ac/Ds pools

An equal amount of every U6 guide in the desired pool (we have tested four to 15 guides; in theory this can be higher if necessary) was combined to obtain a pool at least 40 ng/µl in concentration measured on Nanodrop. This usually required at least 200 ng per guide. If necessary, the pool was adjusted to 40-50 ng/µl with nuclease-free water. 10 µl was transformed into 100 µl Stellar^TM^ Competent Cells (636763, Takara), 1 ml prewarmed SOC medium was added, then incubated at 37°C with shaking for 1 h. For each transformation, two ampicillin agar plates were prewarmed prior to plating. One plate was plated with 100 µl of 1:100 dilution (10 µl of transformation plus 990 µl pre-warmed SOC). The remaining undiluted transformation was pelleted at 4000 ***g*** for 10 min at RT and excess SOC removed until ∼200 µl remained for resuspension followed by plating. Following overnight incubation at 37°C, the Total Colony Yield (no.of colonies×100÷0.1) was determined using the 1:100 plate. To ensure sufficient transformation efficiency, the Total Colony Yield must be at least 1000X greater than the number of guides in the pool (e.g. at least 15 colonies for a pool of 15 guides, in our hands using Stellar cells, we consistently achieved >10 times this rate). To prepare preps, 12 ml of prechilled plain LB broth was dispensed onto the undiluted plate and the bacteria gently scraped into the media followed by careful collection of the slurry into a prechilled Falcon tube. This step was repeated once to ensure thorough collection of colonies. 5 ml of the slurry was used for Miniprep preparation of the final pool (QIAprep Spin Miniprep kit (27104, Qiagen), with the remaining ∼20 ml pelleted, supernatant removed and frozen as back-up for Midiprep. Final pools were quantified using Qubit^TM^ dsDNA HS Assay kit (Q32851, ThermoFisher) and representation of guides assessed by PCR.

#### Microinjection conditions

The following serves as a starting guide and should be adjusted depending on user and microinjector. For Ac/Ds vectors, each embryo was injected with 30 pg of DNA and 24 pg Ac mRNA. For comparison with Tol2 vectors, each embryo was injected with 30 pg of DNA and 24 pg Tol2 mRNA, or 150 pg of DNA and 50 pg Tol2 mRNA (lethality ∼50%). For CRISPRi experiments, 200 pg of sgRNA pool per condition (experimental and scrambled) and 24 pg Ac mRNA were injected per embryo. All microinjections were performed by injecting ∼2 nl into the blastula of one-cell-stage embryos within 5 to 20 min post fertilisation.

### Ac/Ds-U6a:sgRNA vector versus *in vitro*-transcribed sgRNA RT-PCR

Total RNA was extracted from pools of 11 microinjected embryos per condition (vector, or IVT) using RNAqueous-Micro Total RNA Isolation Kit (AM1931, ThermoFisher). Reverse transcription (RT) was performed using 0.5 µM of *R_sgRNA_scaffold_tail* (Table S3) in a 10 µl reaction (1 µg starting RNA) with SuperScript III Reverse Transcriptase (18080093, ThermoFisher) at 55°C for 60 min. Primary PCR was performed using the following primers: *R_sgRNA* and *F_scrambled1_spacer* (Table S3). In a 20 µl reaction, 0.1 µM per primer was combined with 1 µl of template (reverse transcription reaction) in 1X standard Taq polymerase PCR reaction. Cycling was performed as follows: 95°C 5 min, 35X (95°C 30 s, 55°C 30 s), 68°C 30 s, 12°C hold. Next, secondary PCR was performed using the following primers: *R_nested_sgRNA* and *F_nested_scrambled1_spacer* (Table S3). In a 20 µl reaction, 0.1 µM per primer was combined with 1 µl of template (primary PCR reaction) in 1X standard Taq polymerase PCR reaction. Cycling was performed as follows: 95°C 5 min, 23X (95°C 30 s, 55°C 30 s), 68°C 30 s, 12°C hold. Results were analysed on a single 2% agarose gel with 100 bp ladder.

### Generation of CRISPRi transgenic line

TgBAC(sox10:dCas9-SID4x-2a-Citrine)^ox117^ was generated using BAC recombination followed by Tol2 transgenesis as previously described (Suster et al., 2011; Trinh et al., 2017). Recombination cassettes were amplified from pGEM-T-Easy-HA-NLS-dCas9-NLS-SID4x-TaV-2a-Citrine-FRT-Kan-FRT (Addgene ID 119065) to introduce 50 bp homology arms for recombination into BAC clone DKEY-201F15. The first exon of *sox10* was replaced with the recombination cassette.

### Embryo dissociation and FACS

24 hpf CRISPRi-microinjected embryos (experimental and scrambled) were dechorionated and collected into low-binding microcentrifuge tubes. E3 medium was removed, and embryos washed once with 0.22 µM filter-sterilised Hank's buffer: 1X HBSS (14185052, ThermoFisher), 2.5 mg/ml BSA (A3059, Sigma-Aldrich), 10 mM HEPES (15630056, ThermoFisher). For dissociation, up to 200 embryos were incubated in 600 µl of Dissociation Solution: Papain 0.02 U/µl (10108014001, Sigma-Aldrich), ROCK inhibitor 10 µg/ml (A3008, Generon UK), DNaseI 1 mg/ml (10104159001, Sigma-Aldrich) in sterile Hank's buffer. Samples were heatblock-incubated at 37°C and triturated every 5 min using a p200 low-binding tip for no longer than 20 min in total. Once dissociation was complete, reactions were stopped by adding each sample to 4 ml of Stop Solution: DNaseI 1 mg/ml, ROCK inhibitor 10 µg/ml in sterile Hank's buffer. Each sample was triturated gently with a glass serological pipette and passed through a 40 µM cell strainer into a fresh 50 ml Falcon tube. Cells were pelleted at 500 g for 7 min at 4°C. Supernatants were carefully removed until 500 µl remained. 0.5 µl eBioscience™ Fixable Viability Dye eFluor™ 780 (65086514, ThermoFisher) was added, cells gently resuspended with a low-binding tip, and transferred into 5 ml polystyrene round tubes (7340443, VWR) for FACS. Cell sorting was performed on BD Aria III or Fusion with 100 µM nozzle. Citrine+ cells were collected into 100 µl Hank's buffer in normal binding microcentrifuge tubes and placed on ice. Collected cells were immediately pelleted at 500 g for 5 min at 4°C and Hank's buffer carefully removed. Total RNA was extracted and DNaseI-treated using RNAqueous-Micro Total RNA Isolation Kit (AM1931, ThermoFisher) under strict RNase-free conditions. Yield was quantified using Qubit RNA HS Assay kit (Q32852, ThermoFisher) for downstream applications.

### cdh7a and pdgfra qRT-PCR

Equal amounts of RNA per condition (experimental and scrambled) were used as input for cDNA synthesis. cDNA synthesis was performed according to manufacturer's protocol using GoScript Reverse Transcription Mix, Oligo(dT) (A2790, Promega) with -RT controls included. For qPCR, 1 µl of cDNA was used in 10 µl reactions on StepOnePlus Real-Time PCR System and Software v2.3 (ThermoFisher). TaqMan probes (ThermoFisher) used were *Dr03130102_m1* (*cdh7a*), *Dr03086868_m1* (*pdgfra*), and *Dr03436842_m1* (*gapdh*) as endogenous control. Probes were used in combination with TaqMan Fast Advanced Master Mix (4444556, ThermoFisher). Results were analysed using R library *qpcR* version 1.4-0 by fitting to sigmoidal model (Ritz and Spiess, 2008). Code walkthrough of the analysis is available on https://vchongmorrison.github.io/zfCRISPRi.

### Whole-mount immunohistochemistry

For fixation, 4% formaldehyde was freshly prepared using Pierce™ 16% Formaldehyde (w/v), Methanol-free (28906, ThermoFisher) and calcium-/magnesium-free PBS pH 7.4 (10010001, ThermoFisher). Embryos were fixed for 1 h at RT on a nutator. Fixed embryos were dehydrated by incubation in 1:1 ratio methanol/PBS+0.8% Triton X-100 (PBSTr), followed by storage (at least overnight up to 6 months) in 100% methanol at −20°C. After rehydration from 100% methanol to PBSTr, embryos were blocked in 10% normal goat serum, 1% DMSO in PBSTr for at least 1 h at RT. Primary antibodies were mixed with fresh blocking solution and added to embryos for overnight incubation on nutator at 4°C: chicken anti-GFP (ab13970, Abcam) and rabbit anti-zfSox10 (GTX128374, GeneTex) at 1:200 dilution each. Following extensive washing with PBSTr, secondary antibodies were mixed with fresh PBSTr and added to embryos for incubation on nutator at RT for 4 h: donkey anti-rabbit 568 nm (A10042, ThermoFisher) and goat anti-chicken 647 nm (A21449, ThermoFisher) at 1:400 dilution each. Embryos were washed extensively in PBSTr and Hoechst 33258 (H3569, ThermoFisher) added at 1:1000 dilution (to label nuclei) prior to confocal imaging.

### Hybridization Chain Reaction v3.0

HCR v3.0 (Choi et al., 2018) DNA probes to detect sense and antisense *sox9a* and *foxd3* transcripts, as well as Citrine, were designed using an in-house script (Trivedi and Powell, unpublished). Oligos (Table S3) were ordered desalted and resuspended to 100 µM in RNase-free water from Integrated DNA Technologies. Eight probe pairs and 10 probe pairs were selected to detect sense and antisense transcripts, respectively. Probes were pooled by target and adjusted with RNase-free water to 1 µM working concentration. HCR was performed according to open source modified protocol (Bruce et al., 2021) with the following experimental conditions: Permeabilisation with Detergent Solution for 30 min (no Proteinase K treatment), hybridisation with 16 µl of 1 µM per probe cocktail in 500 µl Probe Hybridisation Buffer. RNA detection and amplification were performed with the following initiator-fluorophore combination: foxd3_coding B3-Alexa647, foxd3_antisense B4-Alexa488, sox9a_coding B1-Alexa647, sox9a_antisense B2-Alexa488. Hoechst 33258 (H3569, ThermoFisher) was added at a 1:1000 dilution (to label nuclei) prior to confocal imaging.

### Confocal microscopy imaging

Live embryos were mounted in 1% low melting point agarose with 0.5 g/L tricaine and kept hydrated with E3 medium containing 0.5 g/L tricaine throughout. Fixed and immunostained samples were mounted in a similar fashion with PBS instead of E3/tricaine. Imaging was performed on Zeiss LSM 780 Inverted using EC Plan-Neofluar 10×/0.30 WD=5.2 mm objective. HCR samples were flat-mounted dorsal-side up and imaged on Zeiss LSM 780 Upright using W Plan-Apo 20×/1.0 DIC VIS-IR WD=1.8 (cover glass corrected) objective. All images were acquired using ZEN 2011 (Black Edition) software.

### Strand-specific RNAseq

25 to 50 ng of total RNA (depending on yield and quality) were used as input for ribo-depletion followed by strand-specific RNAseq library preparation according to manufacturer's protocol using KAPA RNA HyperPrep Kit with RiboErase (HMR) (KK8560, Roche). RNA fragmentation was performed at 94°C for 6 min. Libraries were ligated with 1.5 µM NEBNext Adaptor (E7337A, NEB) followed by amplification/indexing with 10 µM per oligo from NEBNext Multiplex Oligos for Illumina Set 1 (E7335, NEB) for 15 cycles. Fragment sizes of final libraries were assessed using D1000 ScreenTape assay and reagents (5067-5582/3, Agilent). Libraries were quantified by qPCR, with size-adjustment, using NEBNext Library Quant Kit for Illumina (E7630, NEB). For sequencing, equal amounts of each library were pooled followed by requantification by qPCR without size-adjustment. 4 nM of the pool with 1% PhiX spike-in was sequenced using NextSeq 500/550 High Output Kit v2.5 (150 Cycles) (20024907, Illumina) on NextSeq500 platform in paired-end mode (2×80 cycles). Reads were trimmed by quality and for presence of adaptors using *cutadapt* v2.10 (Martin, 2011). Transcript quantification was performed with *kallisto* v0.46.1 (Bray et al., 2016) and differential transcript expression analysis performed with *sleuth* v0.30.0 (Pimentel et al., 2017). Gene Ontology (database released 2022-07-01) overrepresentation was performed using PANTHER release 17.0 on *rbioapi* v0.7.7 (GO Biological Process Complete, Fisher's exact test, FDR correction) (Mi et al., 2019; Rezwani et al., 2022; Thomas et al., 2022). Code walkthrough of the analysis is available on https://vchongmorrison.github.io/zfCRISPRi.

## Supplementary Material

10.1242/biolopen.059995_sup1 Supplementary information
