# Supplementary material for: Ac/Ds transposition for CRISPR/dCas9-SID4x epigenome modulation in zebrafish
Source: Biol Open. 2023 Jun 27;12(6):bio059995. doi: 10.1242/bio.059995 (PMC10320716; doi:10.1242/bio.059995)
Supplement: Supplementary information [file biolopen-12-059995-s1.pdf]

Table S1. Ac/Ds and Tol2 enhancer screening in F0 microinjected embryos.

Enhancers	Expression pattern, stage	Percentage of embryos (+/total)	
		Ac/Ds	Tol2
pax3a_E2	Neural (mid- and hindbrain), 24 hpf	81.9 (68/83)	70.7 (65/92)
pax3a_E5	Dorsal neural tube, 8-10 ss	90.4 (104/115)	56.6 (60/106)
ets1_E5	Cranial and trunk neural crest, 12-14 ss	56.7 (85/150)	39.8 (43/108)
ets1_E4	Cranial and trunk neural crest, 10-12 ss	65.6 (61/93)	40.5 (60/148)
sox10_E2	Cranial neural crest, 26 ss	47.3 (43/91)	27.0 (17/63)
sox10_E5	Otic vesicle ± trunk, 24 hpf	45.2 (57/126)	26.3 (31/118)

Table S2. Antisense transcription RNAseq - differentially expressed transcripts

Click here to download Table S2

Table S3. (A) Plasmids featured in the main study. (B) Plasmids NOT featured in the main study.

Click here to download Table S3

Table S4. (A) Oligo sequences (sgRNAs excluded) (B) Oligo sequences (sgRNAs).

Click here to download Table S4

References

Konermann, S., Brigham, M. D., Trevino, A. E., Joung, J., Abudayyeh, O. O., Barcena, C., Hsu, P. D., Habib, N., Gootenberg, J. S., Nishimasu, H., et al. (2015). Genome-scale transcriptional activation by an engineered CRISPR-Cas9 complex. *Nature* **517**, 583–8.

Zalatan, J. G., Lee, M. E., Almeida, R., Gilbert, L. A., Whitehead, E. H., la Russa, M., Tsai, J. C., Weissman, J. S., Dueber, J. E., Qi, L. S., et al. (2015). Engineering complex synthetic transcriptional programs with CRISPR RNA scaffolds. *Cell* **160**, 339–350.
